# Supplementary material for: Deceptive orbital confinement at edges and pores of carbon-based 1D and 2D nanoarchitectures
Source: Nat Commun. 2024 Feb 5;15:1062. doi: 10.1038/s41467-024-45138-w (PMC10844643; doi:10.1038/s41467-024-45138-w)
Supplement: Supplementary file 1 — Supplementary Information [file 41467_2024_45138_MOESM1_ESM.pdf]

Supplementary Information:

Deceptive orbital confinement at edges and pores of carbon-based 1D and 2D nanoarchitectures

Ignacio Piquero-Zulaica[*,1,†], Eduardo Corral-Rascón[1,†], Xabier Diaz de Cerio[2,†], Alexander Riss[*,1], Biao Yang[1], Aran Garcia-Lekue[*,2,3], Mohammad A. Kher-Elden[4], Zakaria M. Abd El-Fattah[4], Shunpei Nobusue[5], Takahiro Kojima[5], Knud Seufert[1], Hiroshi Sakaguchi[*,5], Willi Auwärter[1], and Johannes V. Barth[1]

[1] Physics Department E20, TUM School of Natural Sciences, Technical University of Munich, James-Franck-Straße 1, D-85748 Garching, Germany
[2] Donostia International Physics Center (DIPC), Paseo Manuel de Lardizabal 4, E-20018 Donostia-San Sebastian, Spain
[3] Ikerbasque, Basque Foundation for Science, 48013, Bilbao, Spain.
[4] Physics Department, Faculty of Science, Al-Azhar University, Nasr City, E-11884, Cairo, Egypt
[5] Institute of Advanced Energy, Kyoto University, Uji, 611-0011, Kyoto, Japan
[†] These authors contributed equally to this work: Ignacio Piquero-Zulaica, Eduardo Corral-Rascón, Xabier Diaz de Cerio

This file discusses the following topics related to the main manuscript:

Supplementary Note 1: Oligomer formation on Au(111)
Supplementary Note 2: g-GNR formation on Au(111)
Supplementary Note 3: Electronic properties of g-GNRs
Supplementary Note 4: NPG formation on Au(111) and electronic properties
Supplementary Note 5: Fourier transform of the 7-AGNR, g-GNR and NPG VBs and CBs wave functions
Supplementary Note 6: Height dependent dI/dV maps for g-GNR and NPG CBs
Supplementary Note 7: Fourier transform of VBs and CBs wave functions, band structure and LDOS map simulations of double-void and divacancy containing nanoarchitectures
Supplementary References

Supplementary Note 1: Oligomer formation on Au(111)

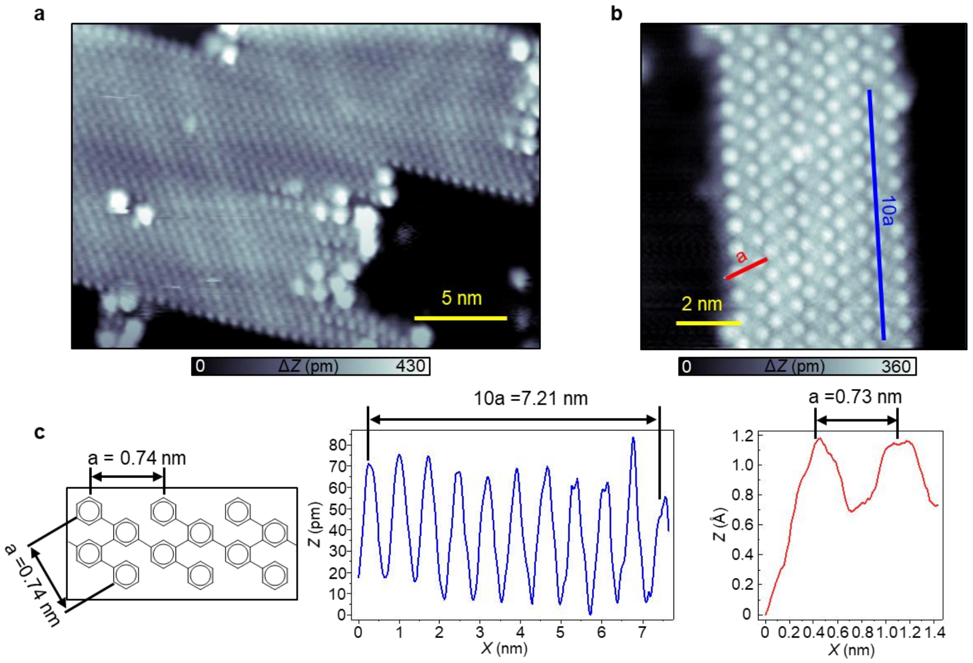

Supplementary Figure 1: **STM characterization of the oligomer chains upon DBQP deposition on Au(111) held at 250 ºC**. (a) STM image (I = 5 pA; V = 300 mV) of a closed-packed oligomer island formed at low surface coverage. *π*-*π* interactions between neighboring oligomers most probably stabilize them into islands. The interaction with the substrate is expected to be weak since the herringbone reconstruction is not significantly altered nor lifted. (b) Close up STM image (I = 5 pA; V = 300 mV) of a close-packed oligomer island. (c) Chemical model of the oligomer chain and line profiles (in blue and red) extracted from panel (b) evidence the expected C–C bond-based oligomer.

Supplementary Note 2: g-GNR formation on Au(111)

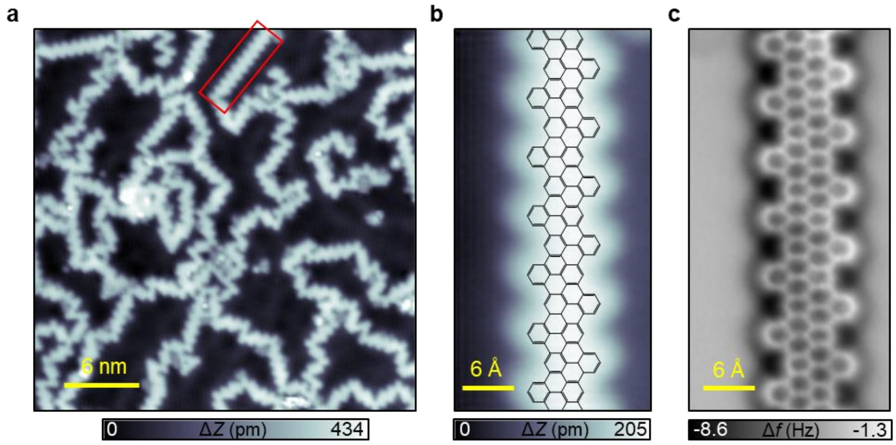

Supplementary Figure 2: **STM and nc-AFM characterization of g-GNRs on Au(111)**. (a) Long range STM image (I = 8 pA; V = 50 mV) of g-GNRs after oligomer cyclodehydrogenation at 500 ºC. Cross-coupled and defective g-GNRs are commonly observed, coexisting with some intact and well-defined g-GNRs segments. (b) High-resolution STM image (I = 8 pA; V = 50 mV) of an intact g-GNR segment with six unit cells in length. The overlaid chemical model accurately matches the expected g-GNR morphology with its characteristic gulf edges. (c) High-resolution nc-AFM image of the same g-GNR acquired with a CO functionalized tip.

Supplementary Figure 3 summarizes some of the defects identified with nc-AFM measurements after annealing the sample to 500 ºC. Terphenyl junctions (Motif A) between g-GNR segments are quite abundant and are suggested to form due to the flipping of the oligomer segments during the g-GNR planarization process [panel (a)]. Carbon atom rearrangements and g-GNR cross-coupling events may also be responsible for the other defect types observed [see Motifs B and C in panels (b-d)]. A more detailed study of the types of defects and their formation mechanisms is beyond the scope of this manuscript.

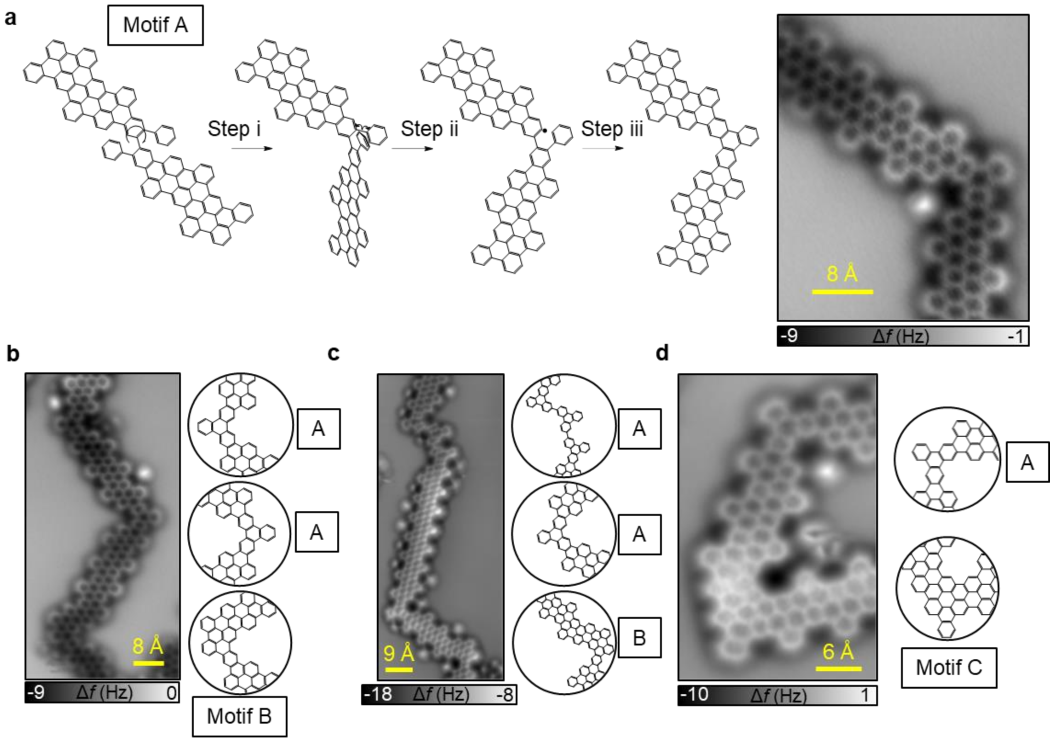

Supplementary Figure 3: **Characterization of the g-GNR defects with nc-AFM**. (a) Suggested formation mechanism of a terphenyl defect (Motif A) between g-GNR segments. (b-d) Additional defects commonly observed after g-GNR synthesis (Motifs B and C).

Supplementary Note 3: Electronic properties of g-GNRs

Supplementary Note 3.1: STM/STS characterization of g-GNRs with increasing lengths

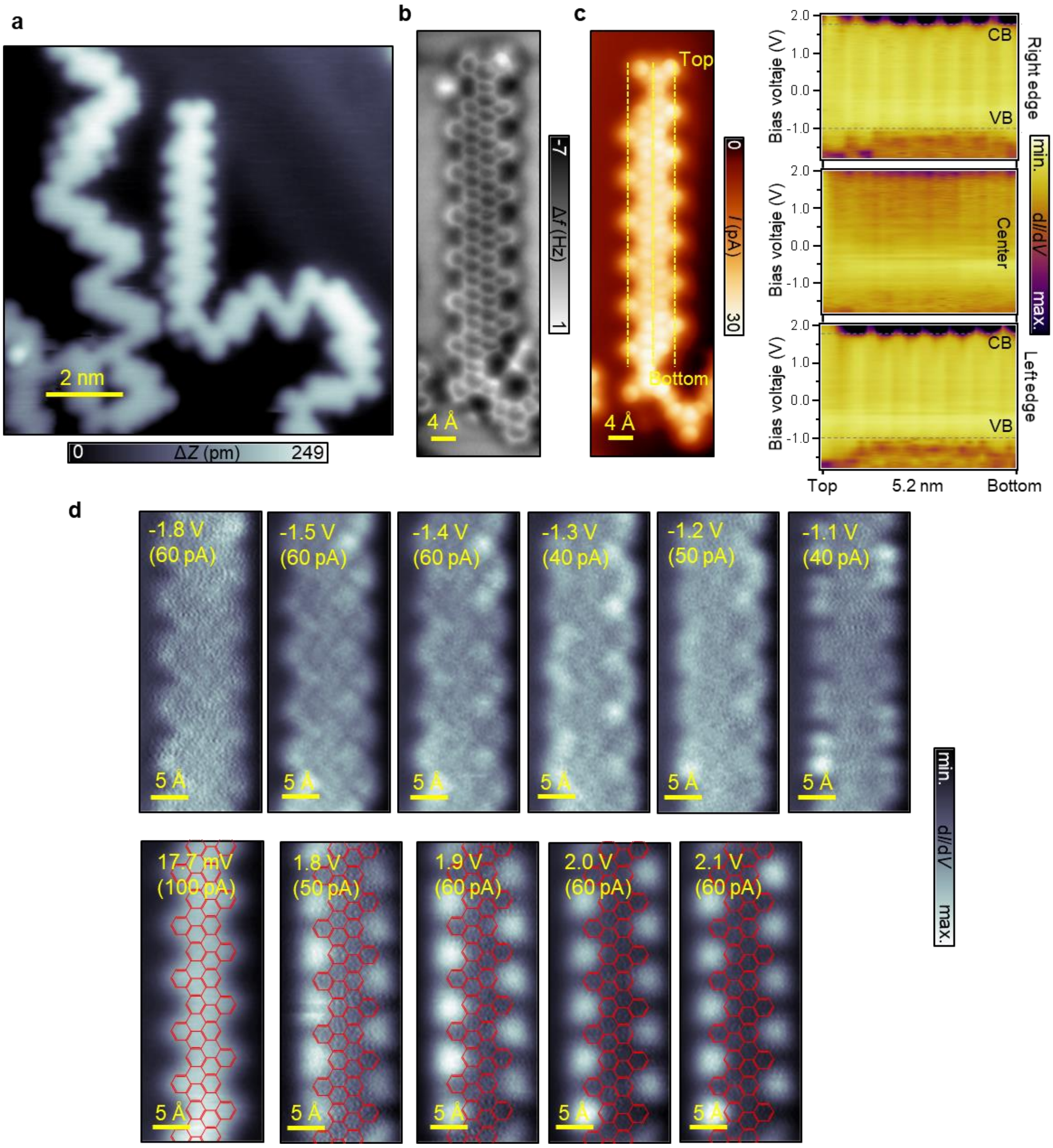

Supplementary Figure 4: **Electronic properties of a five-unit cell long g-GNR (CO-tip)**. (a) Overview STM image (I = 6 pA; V = 500 mV) with a g-GNR segment surrounded by defective GNR segments. (b) Nc-AFM image of the g-GNR segment highlighting its well-defined structure and triphenylene terminations at both sides. (c) BR-STM image (V= -8.3 mV) and LDOS intensity maps of line dI/dV point spectra acquired at both edges and center of the g-GNR (I= 100 pA; V= 2 V). The onsets of VB and CB can be clearly identified and a ≈2.8 V wide-bandgap is obtained. (d) Set of constant-height dI/dV maps acquired for VBs, within the gap and CBs.

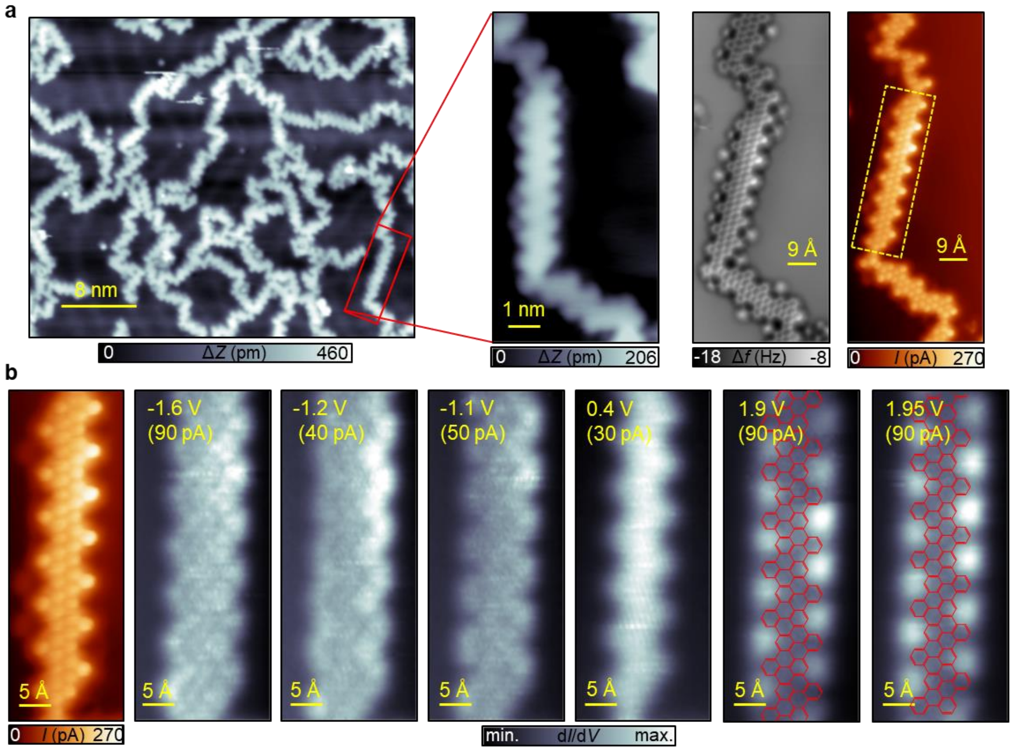

Supplementary Figure 5: **Electronic properties of a six-unit cell long g-GNR linked to g-GNR segments via triphenylene junctions (CO-tip)**. (a) (from left to right) Overview STM image (I = 6 pA; V = 100mV) where the six-unit cell long g-GNR segment is identified (red rectangle). Nc-AFM and BR-STM (V = -13.3 mV) images corroborate its atomically precise and well-defined structure. Bright and dim dots attached to the gulfs can be clearly seen in AFM, which we tentatively attribute to CO and Br species. (b) Set of constant-height dI/dV maps acquired for VBs, within the gap and CBs.

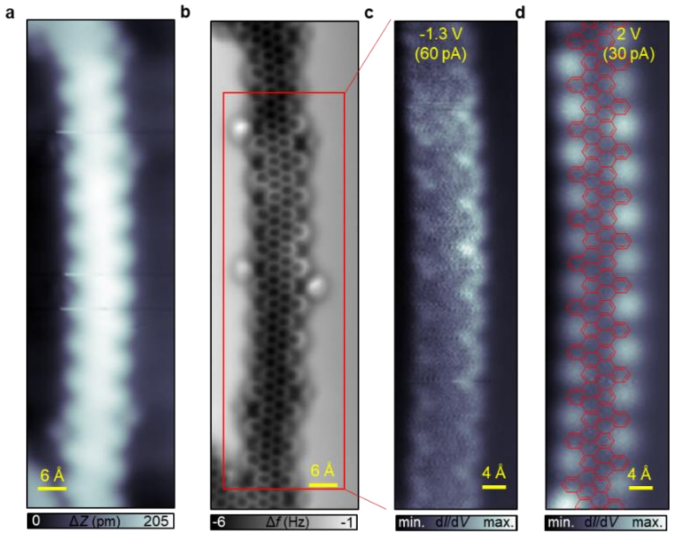

Supplementary Figure 6: **Electronic properties of a twelve-unit cell long g-GNR crossing the herringbone (CO-tip)**. (a) High-resolution STM image (I = 6 pA; V = 100 mV) of the g-GNR. (b) Nc-AFM image of the g-GNR confirms its well-defined structure and appears slightly bent, likely due to the corrugation of the herringbone beneath it. (c,d) Constant-height dI/dV maps acquired close to the VB and CB onsets appear homogeneous along the g-GNR, showing no significant effect of the herringbone reconstruction.

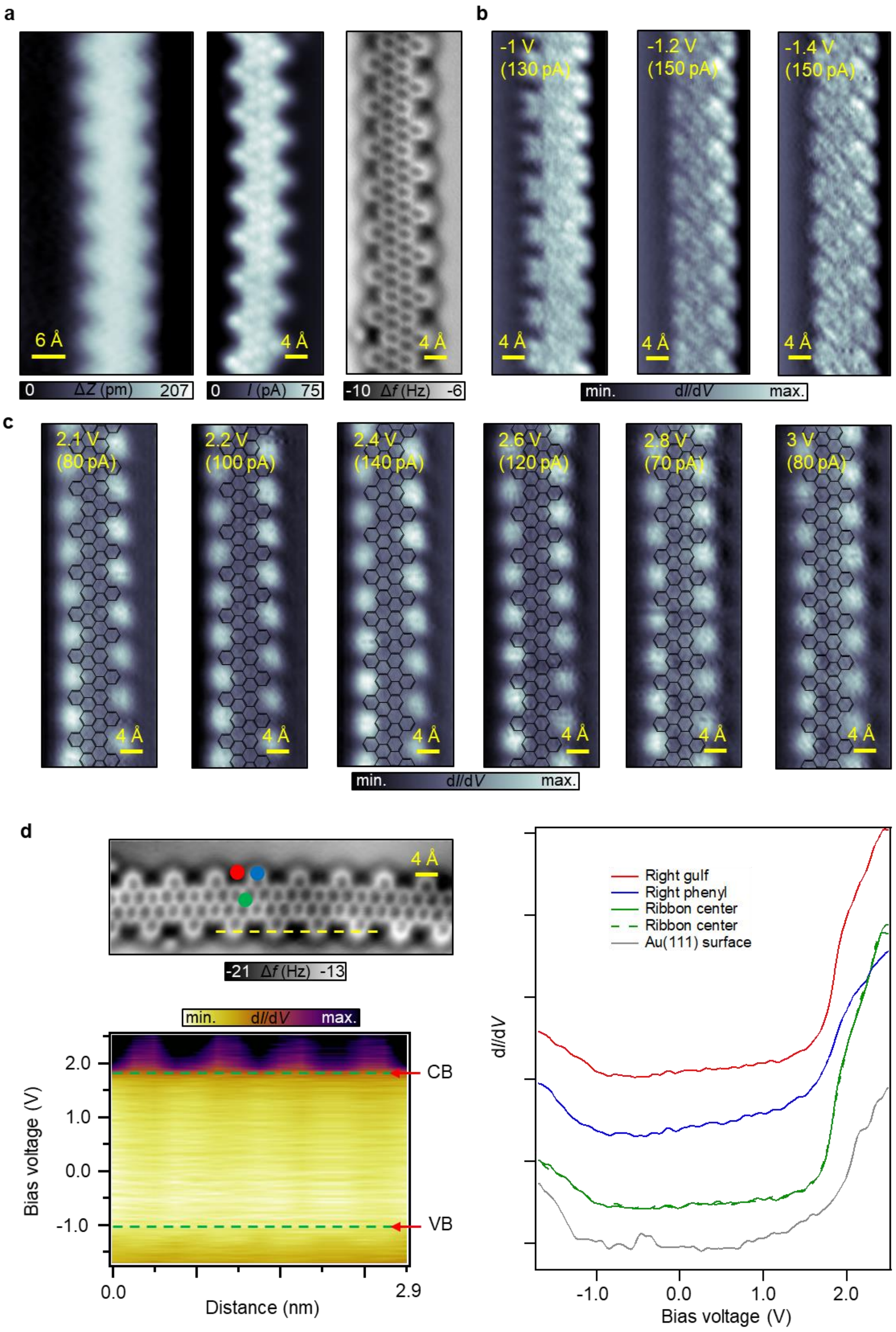

Supplementary Figure 7: **Electronic properties of an eight-unit cell long g-GNR (CO-tip)**. (a) (from left to right) High-resolution STM (*I* = 10 pA; *V* = 100 mV), BR-STM (V = 3 mV) and nc-AFM images of the g-GNR confirm its well-defined structure. (b,c) Set of constant-height d*I*/d*V* maps acquired for VBs and CBs, respectively. For the CBs the maps are extended up to 3 V, beyond which g-GNR and CO-tip integrity are compromised. (d) LDOS intensity map corresponding to a line d*I*/d*V* point spectra (*I* = 100 pA; *V* = 2.5V; open feedback loop) acquired at one edge of the g-GNR and point spectra acquired at different positions of the g-GNR: gulf (red), edge phenyl (blue) and ribbon center (green). Au surface spectrum is shown in grey. Despite the LDOS asymmetry, a ≈2.8 V wide-bandgap can be measured.

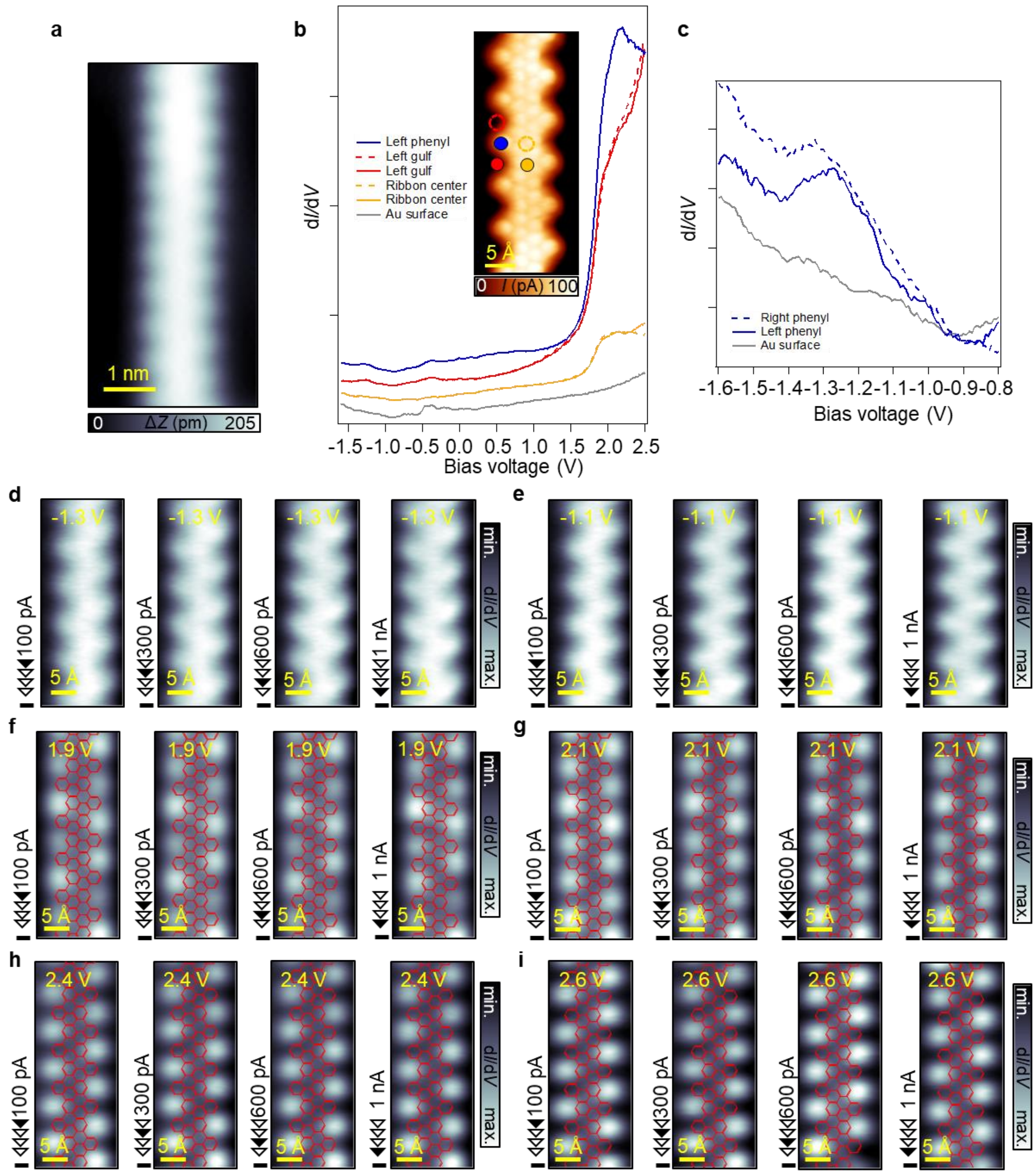

Supplementary Figure 8: **Electronic properties characterization of a ten-unit cell long g-GNR using a metal tip**. (a) STM image of the g-GNR (I = 100 pA; V = 500mV). (b) dI/dV point spectra acquired at different positions of the g-GNR [phenyl (blue), gulf (red), ribbon center (orange)] and Au surface (grey) (I = 70 pA; V = -1.6 V). (c) dI/dV point spectra acquired around the onset of the VB at the edge phenyl positions (blue) and Au surface (grey). (d) Height dependent dI/dV maps acquired for -1.3 V (d), -1.1 V (e), 1.9 V (f), 2.1 V (g), 2.4 V (h) and 2.6 V (i). The height is altered by changing the current set-point stepwise from 100 pA to 1 nA.

Supplementary Note 3.2: DFT band structure, electronic orbitals and LDOS map simulations

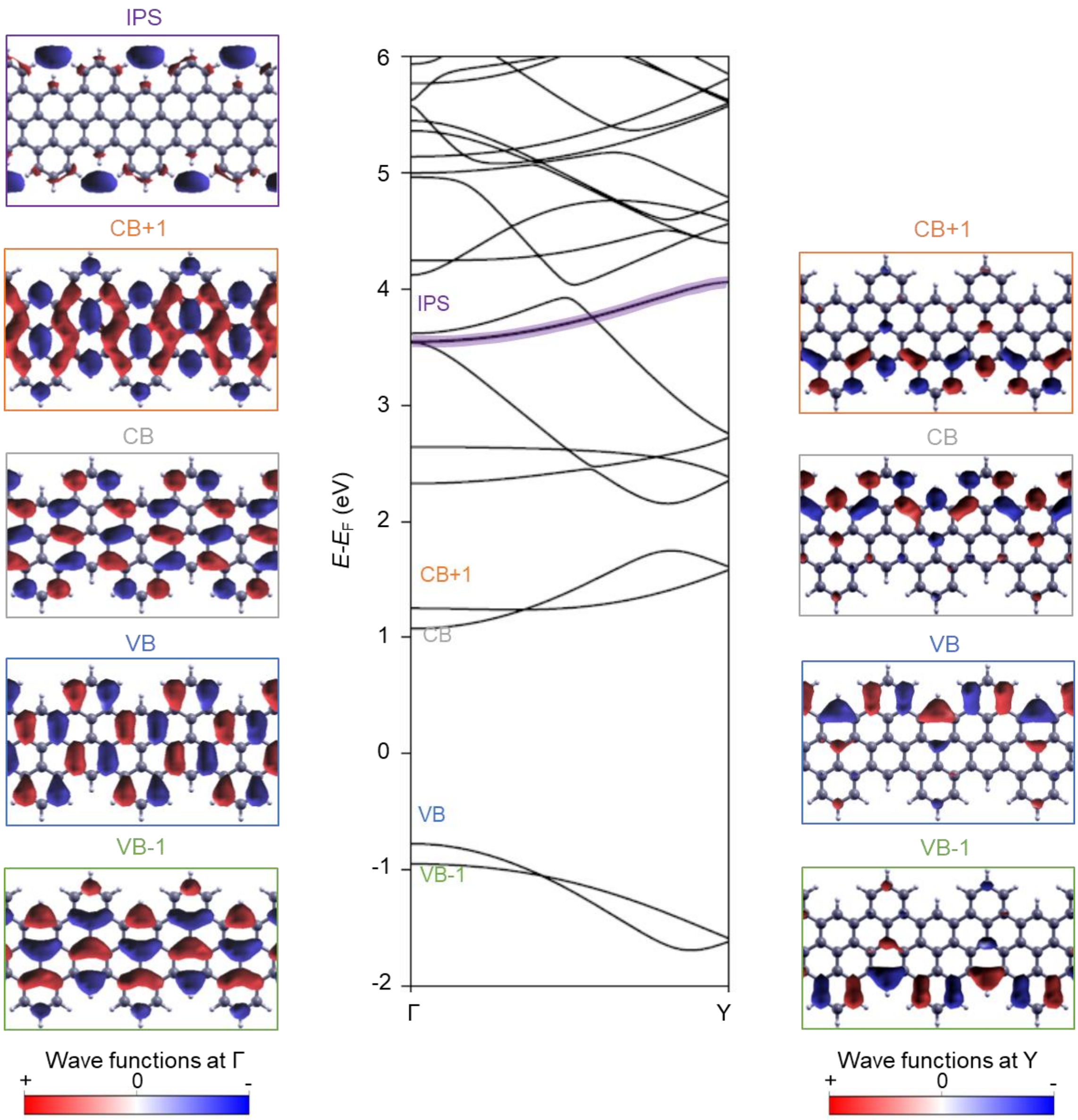

Supplementary Figure 9: **DFT calculated band structure for the g-GNR**. It shows dispersive and braiding VBs and CBs and a semiconducting gap of 1.84 eV. Image potential states (IPS) are identified at higher energies. Such free electron-like IPS are confined at the vacuum side along the g-GNR edge. Frontier wave functions for VB-1 (green), VB (blue), CB (grey) and CB+1 (orange) extracted at the Γ and Y points and wave function for the IPS (purple) extracted at the Γ point. The IPS is the only one that shows wave functions intrinsically confined at the gulfs.

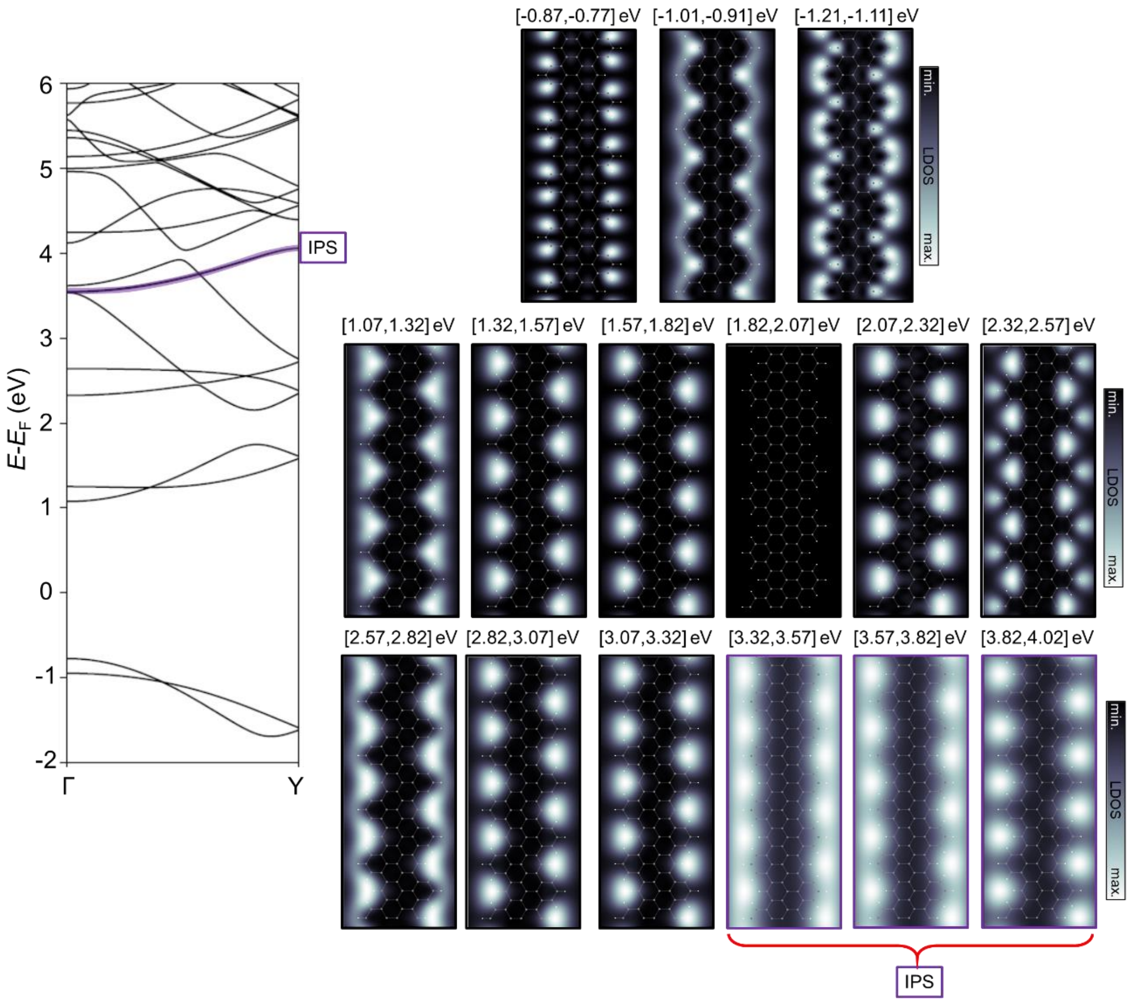

Supplementary Figure 10: **DFT LDOS map simulations of the g-GNRs extracted at a height of *z* = 5 Å.** An energy integration of 100 meV and 250 meV is chosen for occupied and unoccupied states respectively. The VBs show conductance features localized at the phenyl rings (appearing as two dots) and winding along the edges. All the CBs show conductance features confined at the gulf regions, which coincidentally resemble the conductance features of IPS.

Supplementary Note 4: NPG formation on Au(111) and electronic properties

Supplementary Note 4.1: STM/STS and nc-AFM NPG characterization

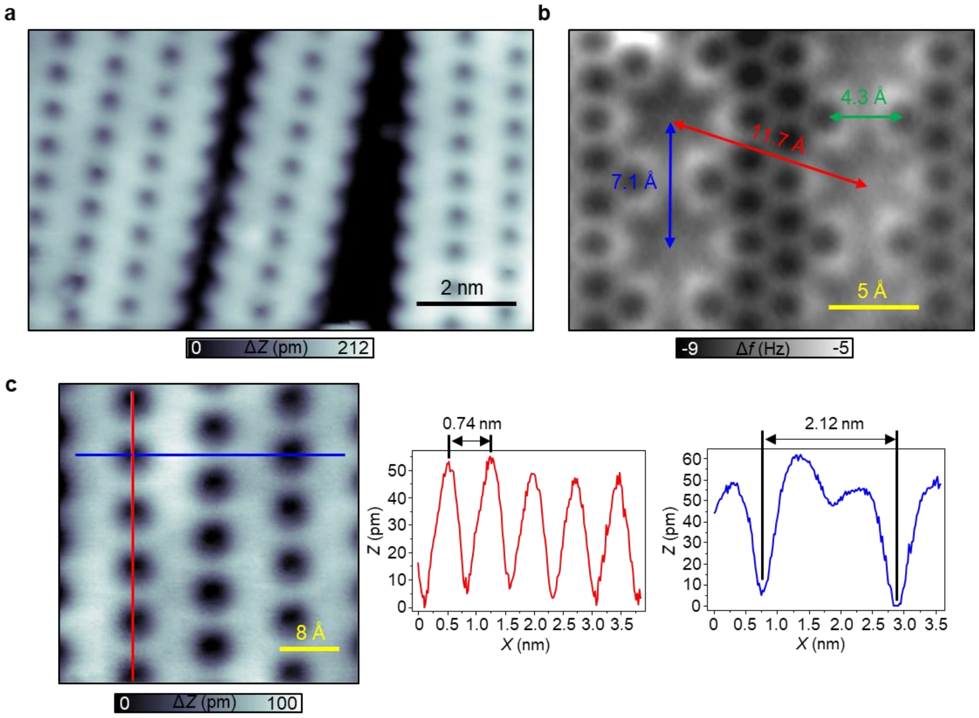

Supplementary Figure 11: **STM and nc-AFM characterization of NPGs**. (a) High-resolution STM image (I = 6 pA; V = 100 mV) containing two, three and four g-GNR fused NPGs. (b) Nc-AFM image of the g-GNR fusing position and nanoporous morphology. The distances extracted confirm the covalent character of the bonds formed upon fusion. (c) High-resolution STM image (I = 6 pA; V = 30 mV) of an NPG. Nanopore periodicities extracted along the main g-GNR direction (red) and across (blue).

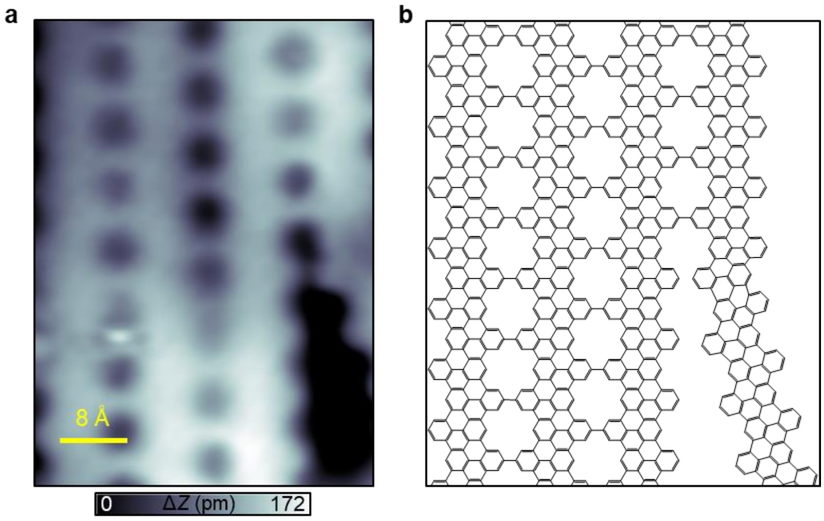

Supplementary Figure 12: **Suggested zipping mechanism for the NPG formation**. (a) High-resolution STM image (I = 5 pA; V= 100 mV) of an incomplete NPG. (b) The chemical model for the imperfect NPG suggests a progressive zipping process upon lateral fusion of g-GNRs.

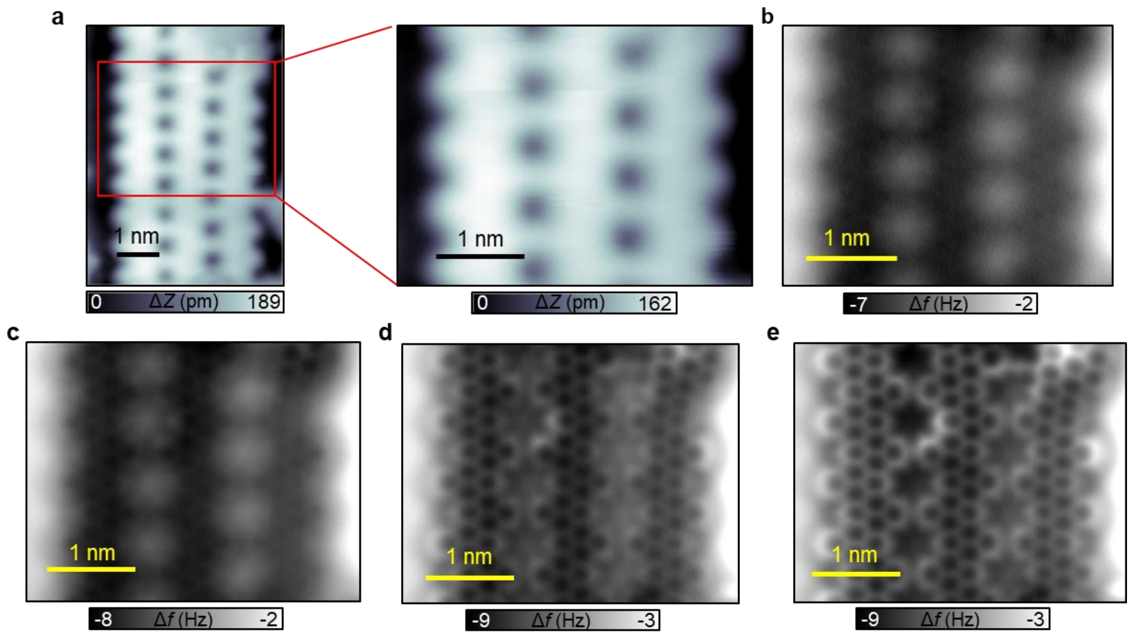

Supplementary Figure 13: **Frequency shift measurements of an NPG with different heights**. (a) High-resolution STM image of an NPG (I = 6 pA; V = 50 mV). (b-e) Nc-AFM images acquired at different heights: 20 pm (c), 60 pm (d) and 80 pm (e) closer than (b). For panels (d) and (e) the bond resolution becomes more pronounced.

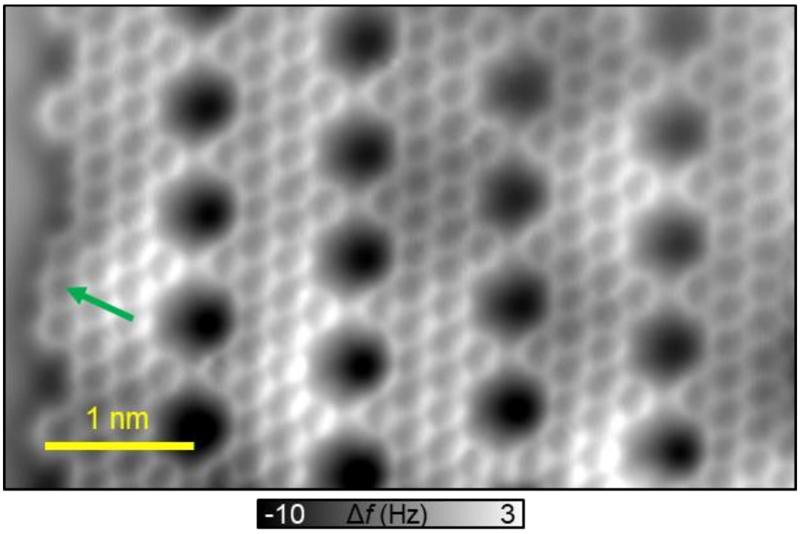

Supplementary Figure 14: **Nc-AFM image of a five g-GNR fused NPG**. Carbon atom rearrangements have taken place at the edge of the NPG (green arrow) and the periodicity and size of the gulfs is altered.

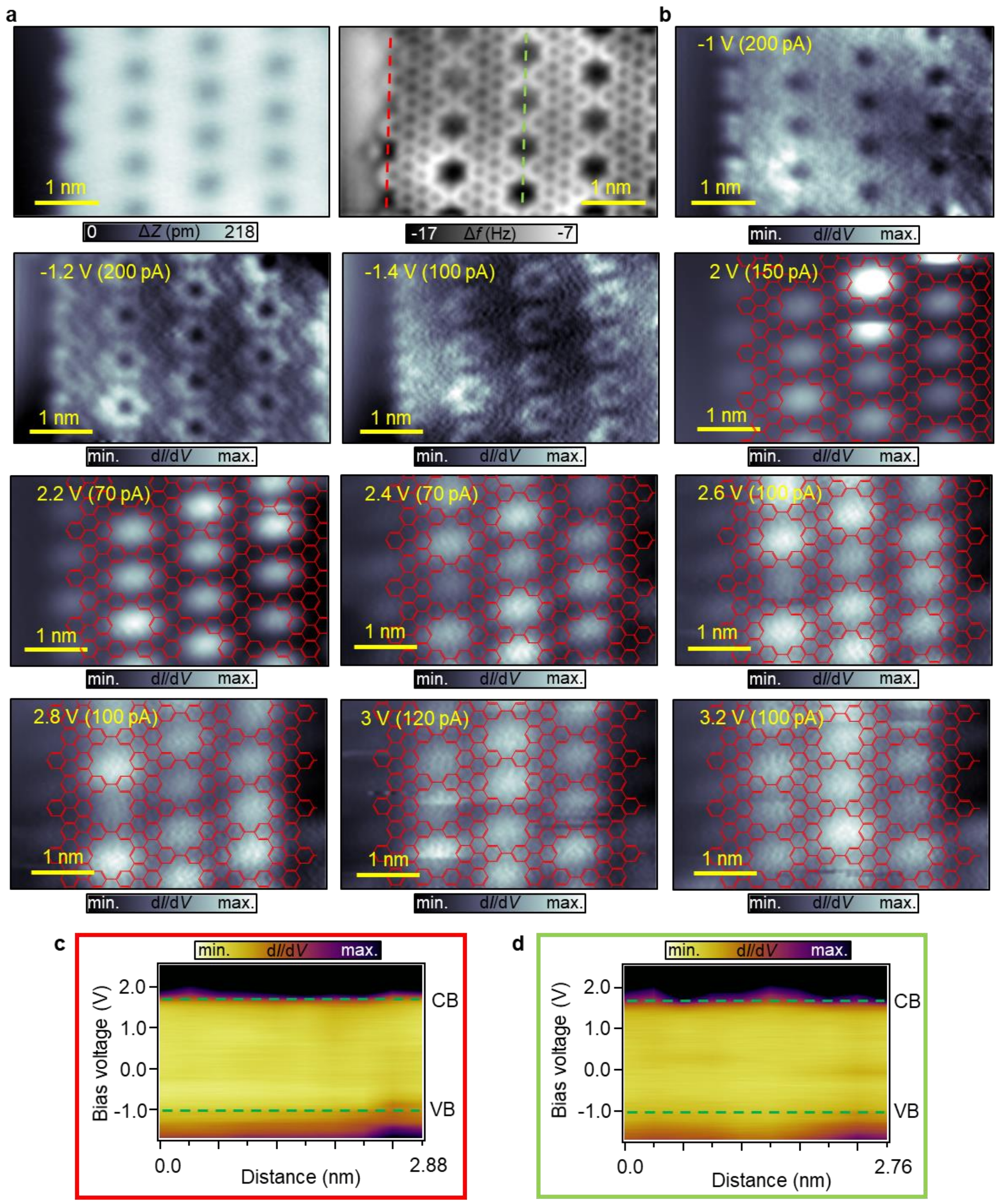

Supplementary Figure 15: **Electronic properties of an NPG (CO-tip)**. (a) High-resolution STM (*I* = 10 pA; *V* = 100 mV) and nc-AFM images of the NPG. The NPG is well-defined and the graphene lattice is unperturbed. (b) Set of constant-height d*I*/d*V* maps acquired for VBs and CBs. For the CBs the d*I*/d*V* maps are measured as high as 3.2 V, beyond which NPG and CO-tip integrity are compromised. (c, d) LDOS intensity maps for d*I*/d*V* line spectra (*I* = 70 pA; *V* = 2.5 V; open feedback loop) acquired at the border and inside the NPG [see red and green dashed lines in panel (a)].

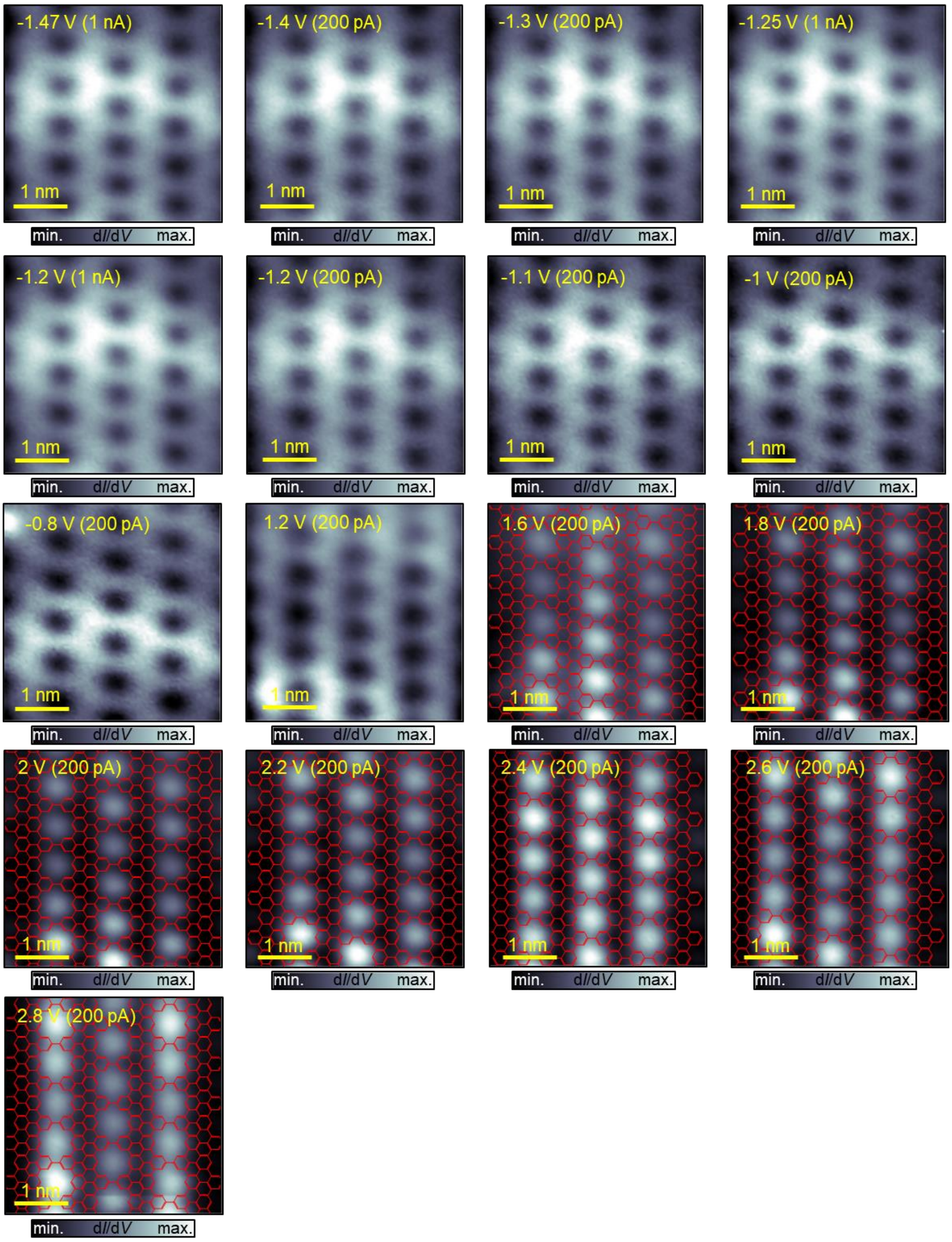

Supplementary Figure 16: **Set of constant-height d*I*/d*V* maps acquired for an NPG with a metal tip**. The bias voltage and current set-points for each d*I*/d*V* map is included in each panel. While the subtle conductance features corresponding to the VBs can not be clearly observed, the CBs are identical to the ones measured with a CO funtionalized tip (see Supplementary Fig. 15).

Supplementary Note 4.2: DFT band structure, molecular-orbital and LDOS map simulations for NPGs

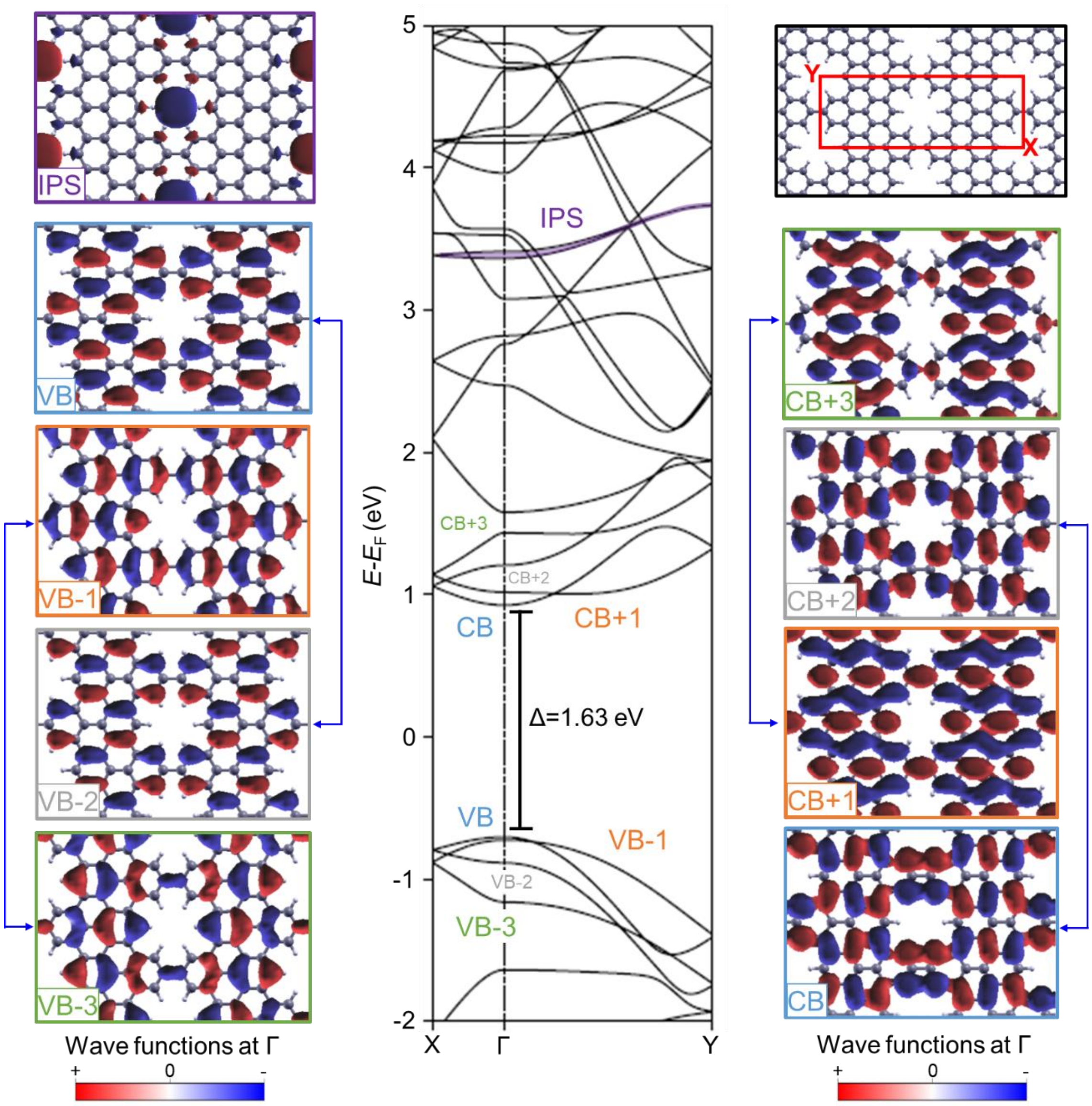

Supplementary Figure 17: **DFT calculated band structure for the NPG**. It shows dispersive VBs and CBs in both longitudinal and transversal directions (i.e., ΓY and ΓX directions) and a semiconducting gap of 1.63 eV. Wave functions for VB-3 (green), VB-2 (gray), VB-1 (orange), VB (blue), CB (blue), CB+1 (orange), CB+2 (gray) and CB+3 (green), as well as for IPS (purple) extracted at the Γ point. Bonding and antibonding states originating from the coupling of individual states in adjacent g-GNRs are indicated with blue arrows. The IPS orbitals are the only ones that appear intrinsically confined at the nanopores.

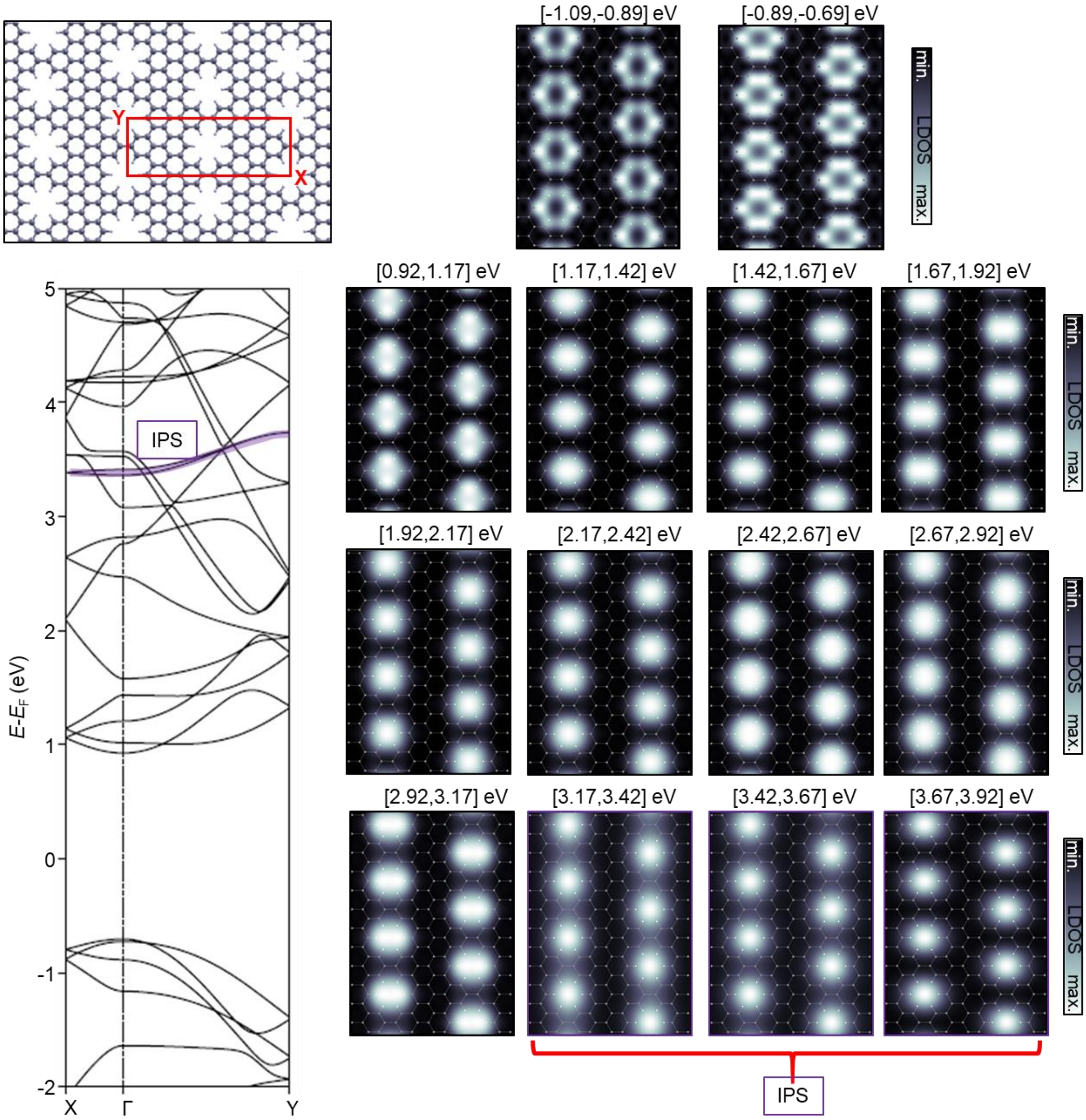

Supplementary Figure 18: **DFT LDOS map simulations of the NPGs extracted at a height of *z* = 5 Å**. An energy integration of 200 meV and 250 meV is chosen for occupied and unoccupied states, respectively. The VBs show conductance features localized around the nanopores (appearing as hexagonal rings). All the CBs show conductance features confined at the nanopores, which coincidentally resemble the conductance features of IPS.

Supplementary Note 4.3: EPWE wave functions, band structure and photoemission intensity simulations for g-GNRs and NPGs

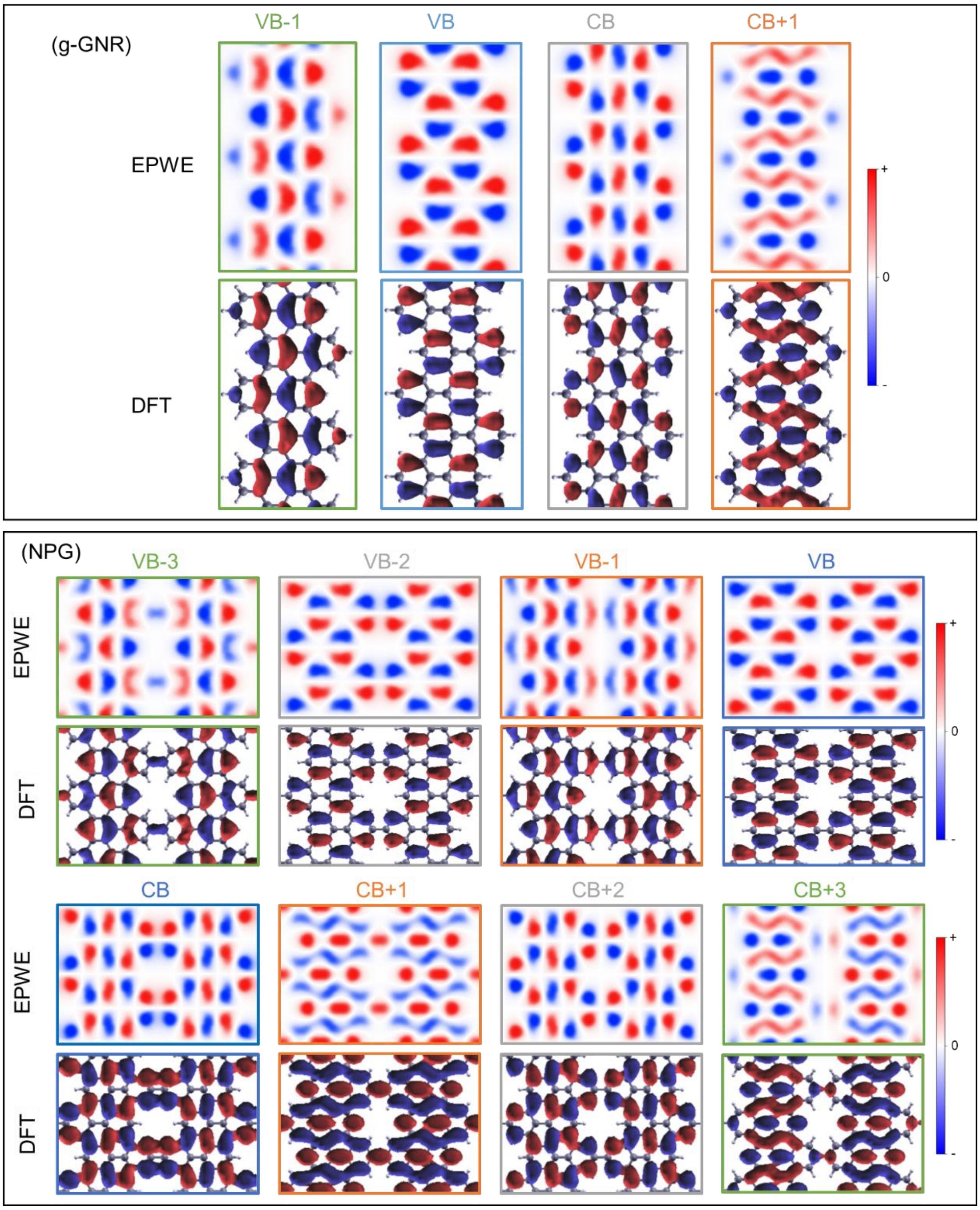

Supplementary Figure 19: **EPWE and DFT g-GNR and NPG orbital calculations**. EPWE wave functions for g-GNRs (top) and NPGs (bottom) in comparison to DFT calculated ones. Wave functions have been extracted at the Γ point. There is a nice agreement between both theoretical approaches.

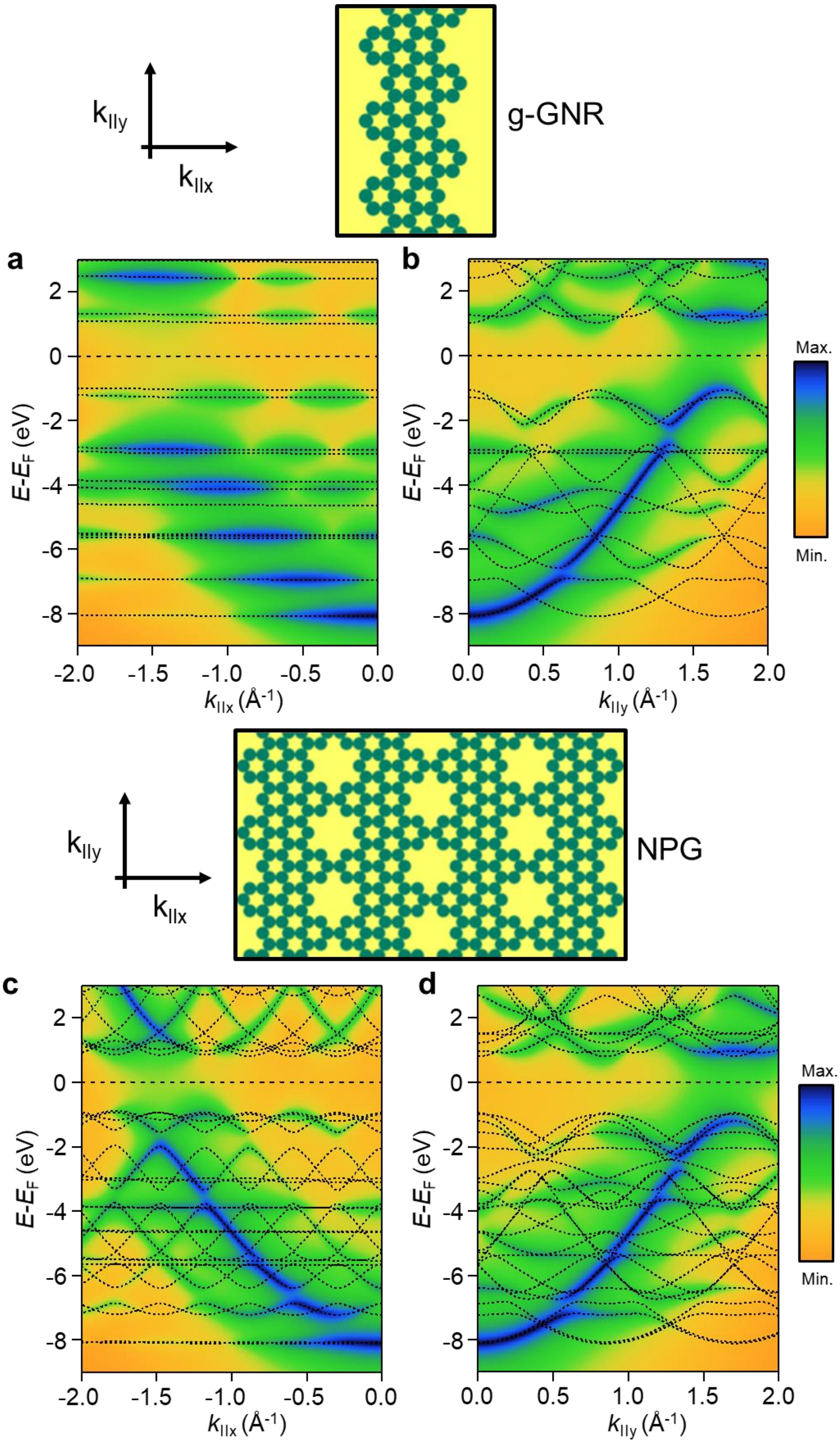

Supplementary Figure 20: **EPWE band structure and photoemission intensity simulations for g-GNRs and NPGs**. The photoemission intensity of the g-GNRs shows dispersive bands in the longitudinal direction, while in the perpendicular direction the bands become non-dispersive. For the NPG case, the electronic properties become highly isotropic since dispersive bands are observed in both longitudinal (along the g-GNRs) and transversal (perpendicular to the g-GNRs) directions.

Supplementary Note 5: Fourier transform of the 7-AGNR, g-GNR and NPG VBs and CBs wave functions:

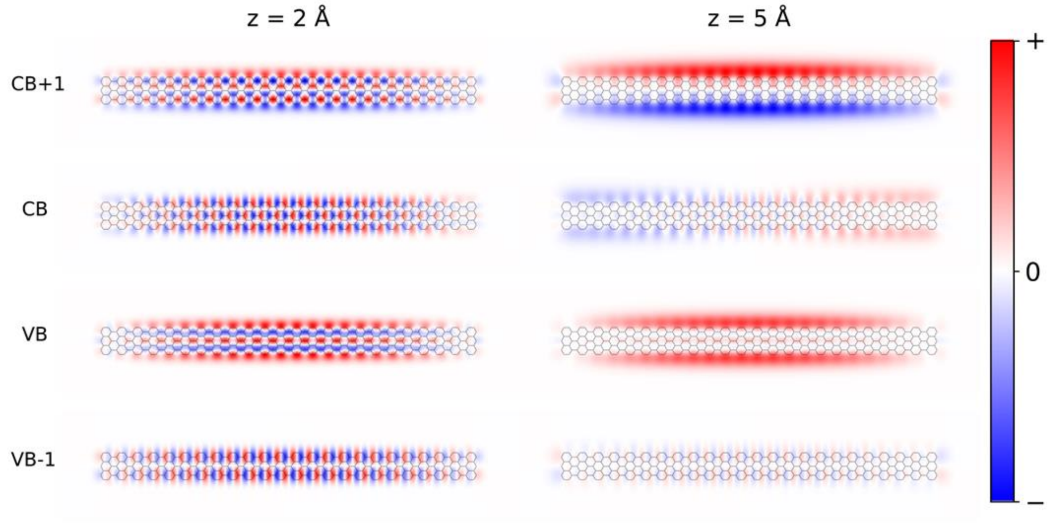

Supplementary Figure 21: **Effect of the tip-sample distance on the decay of 7-AGNR orbitals**. Kohn-Sham orbitals at the band onsets for a 7-AGNR evaluated at z = 2 Å and z = 5 Å. The loss of Fourier components, corresponding to states of high momentum, with increasing probe-sample distance is responsible for the electronic orbital wave function decay and confinement into the GNR edges. These simulations are in good agreement with the ones reported in ref.[1]

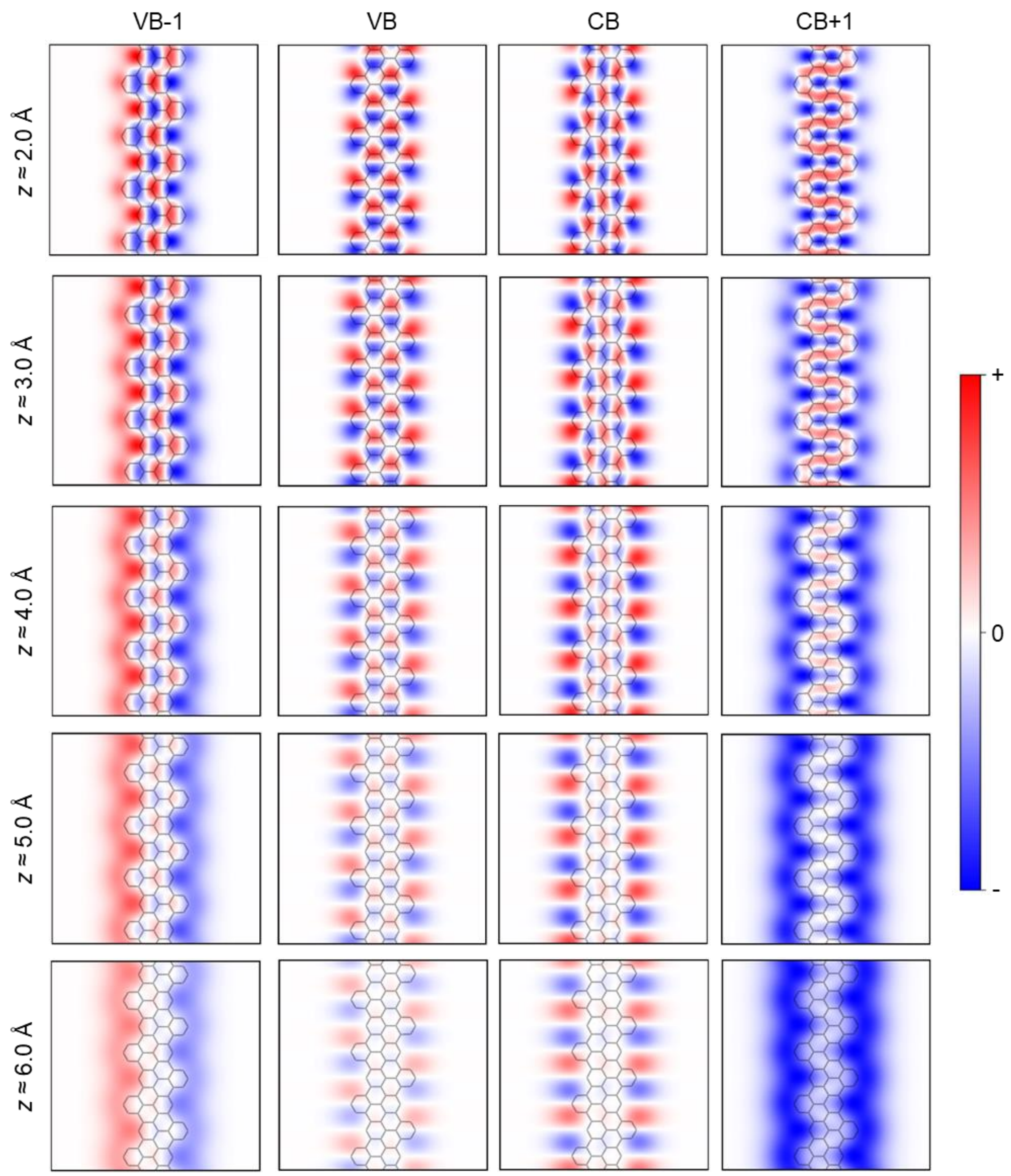

Supplementary Figure 22: **Effect of tip-sample distance on the decay of g-GNR orbitals**. Kohn-Sham orbitals of VB-1, VB, CB and CB+1 at the band onsets (Γ-point) of a g-GNR evaluated from 2 Å to 6 Å.

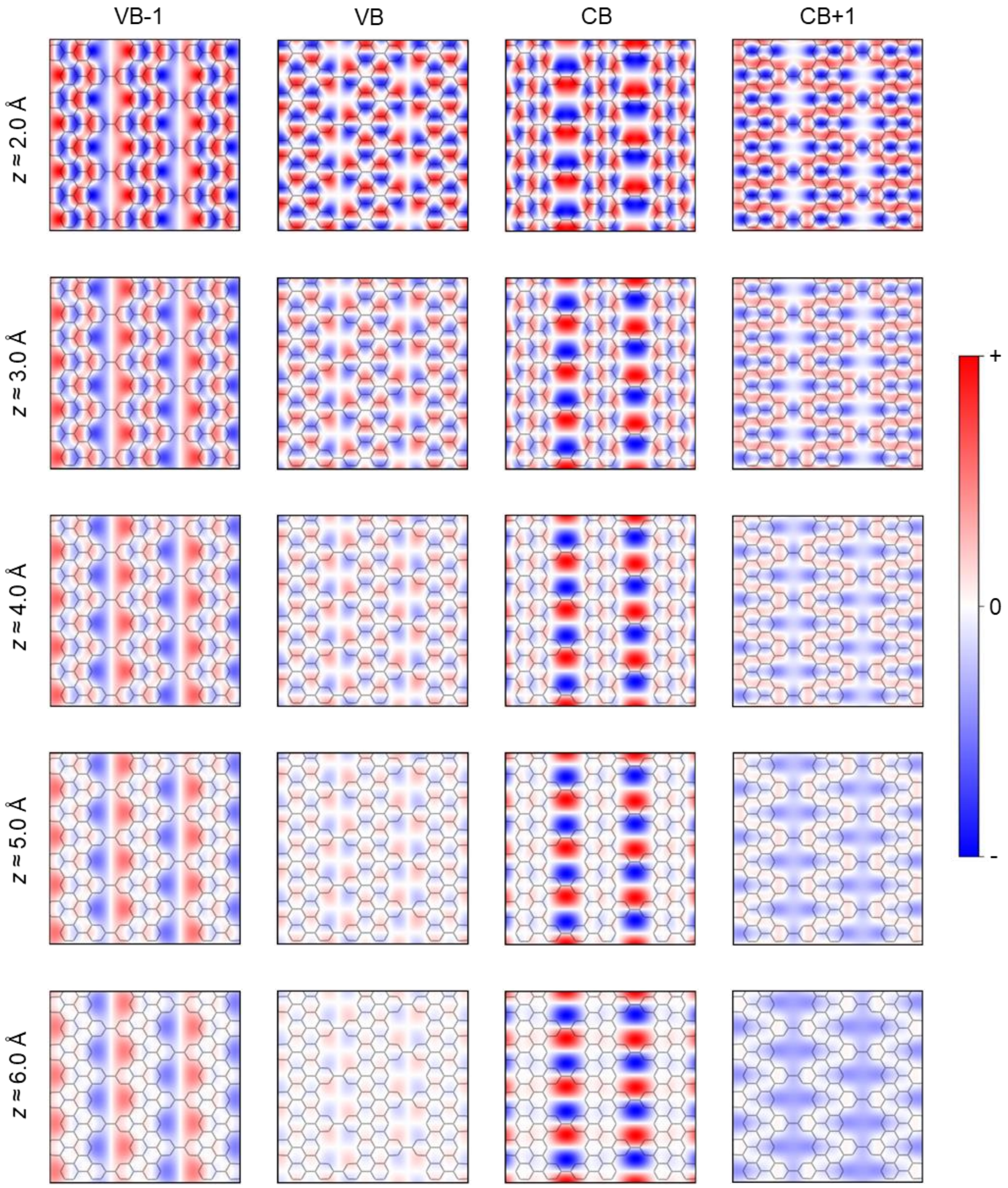

Supplementary Figure 23: **Effect of tip-sample distance on the decay of NPG orbitals**. Kohn-Sham orbitals of VB-1, VB, CB and CB+1 at the band onsets (Γ-point) of a NPG evaluated from 2 Å to 6 Å.

Supplementary Note 6: Height dependent d*I*/d*V* maps for g-GNR and NPG CBs:

In Supplementary Figure 24 we complement the theoretical approach shown in Figure 4 of the main manuscript with d*I*/d*V* maps acquired for the CBs of g-GNR (a-d) and NPG (e-h) with decreasing tip-sample separations. This is done by increasing the current set-point from 100 pA to 1 nA. Such an increase in current causes a decrease of the tip-sample distance by ≈1 Å. As can be observed, the conductance features still remain confined into the edges of the g-GNR (a-d) and nanopores of the NPG (e-h). Therefore, under these circumstances, the STM technique does not allow us to approach the tip sufficiently to the nanoarchitectures so that the conductance features can be observed confined at the carbon backbone (see Supplementary Figures 22 and 23).

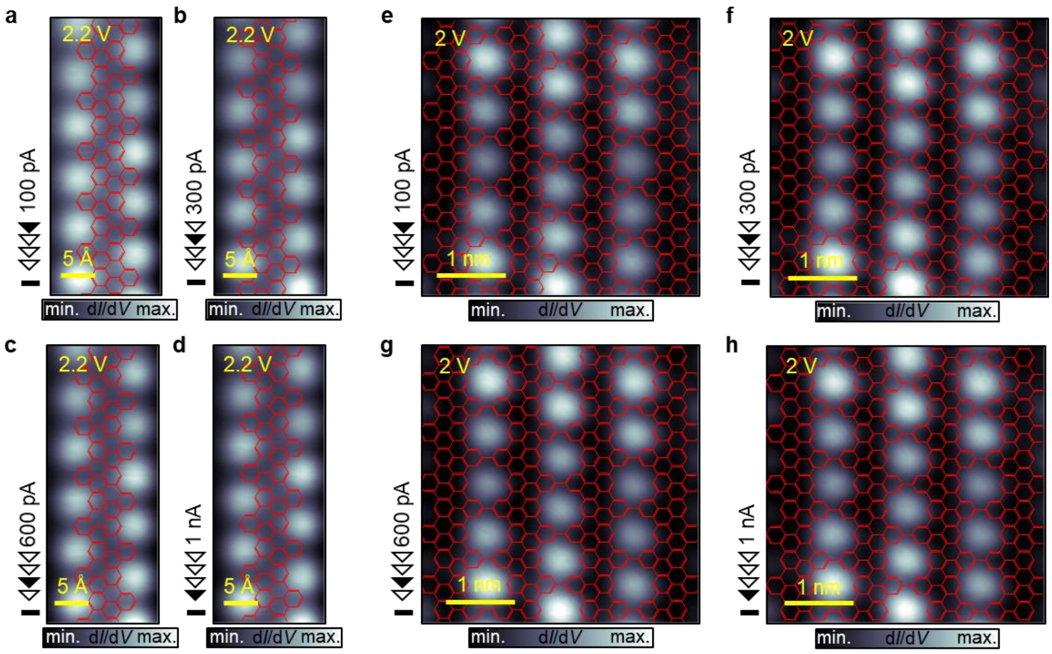

Supplementary Figure 24: Constant height d*I*/d*V* maps acquired with a metal tip for decreasing tip to g-GNR/NPG separation. (a-d) d*I*/d*V* maps obtained for the g-GNR at 2.2 V with increasing current set-points from 100 pA to 1 nA. The STM tip approaches the g-GNR by ≈1 Å. The conductance features remain confined to the g-GNR gulf regions at the edges. (e-h) d*I*/d*V* maps obtained for the NPG at 2 V with increasing current set-points from 100 pA to 1 nA. The conductance features also remain confined at the nanopores.

Supplementary Note 7: Fourier transform of VBs and CBs wave functions, band structure and LDOS map simulations of double-void and divacancy containing nanoarchitectures

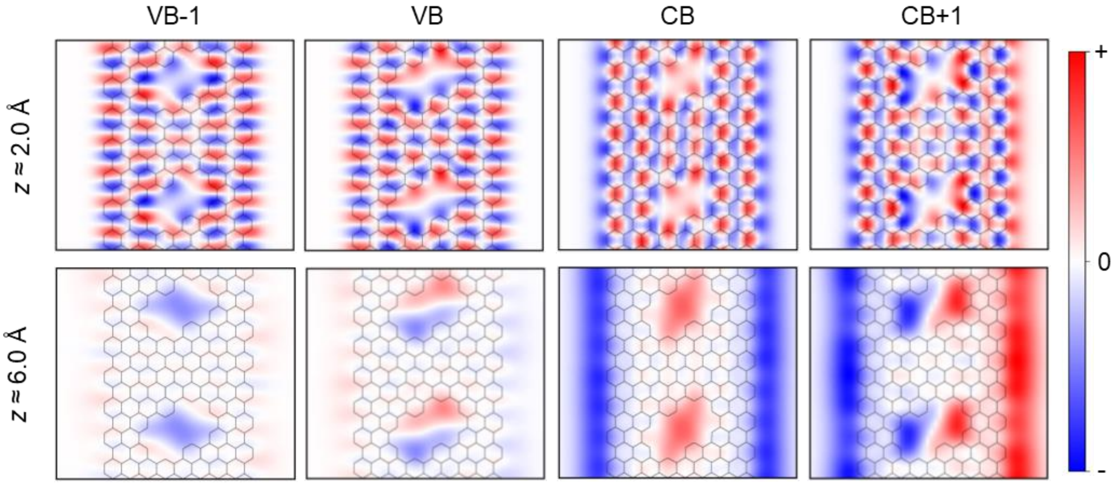

Supplementary Figure 25: **Effect of tip-sample distance on the electronic orbital decay of GNRs with periodically spaced double-voids**. Kohn-Sham orbitals of VB-1, VB, CB and CB+1 at the band onsets (Γ-point) of a GNR evaluated from 2 Å to 6 Å.

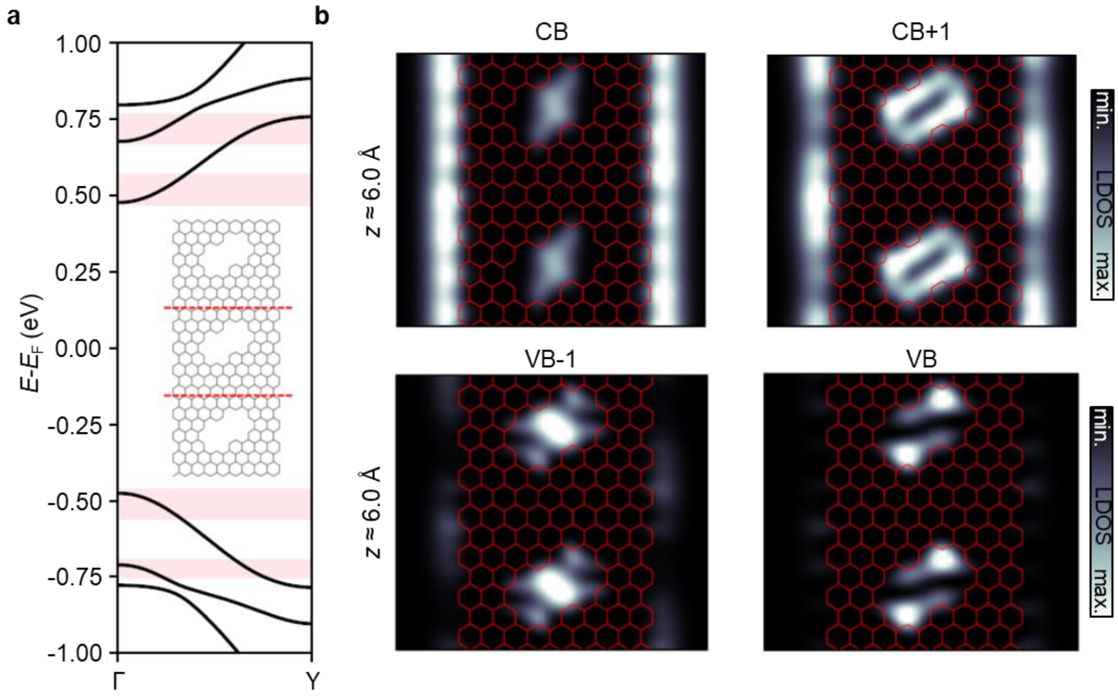

Supplementary Figure 26: **LDOS simulations of double-void GNRs**. Band structure (a) and LDOS map simulations (b) of GNRs with periodically spaced double-voids (evaluated at 6 Å away from the GNR). The electron density decays and is confined into the GNR edges and double-voids.

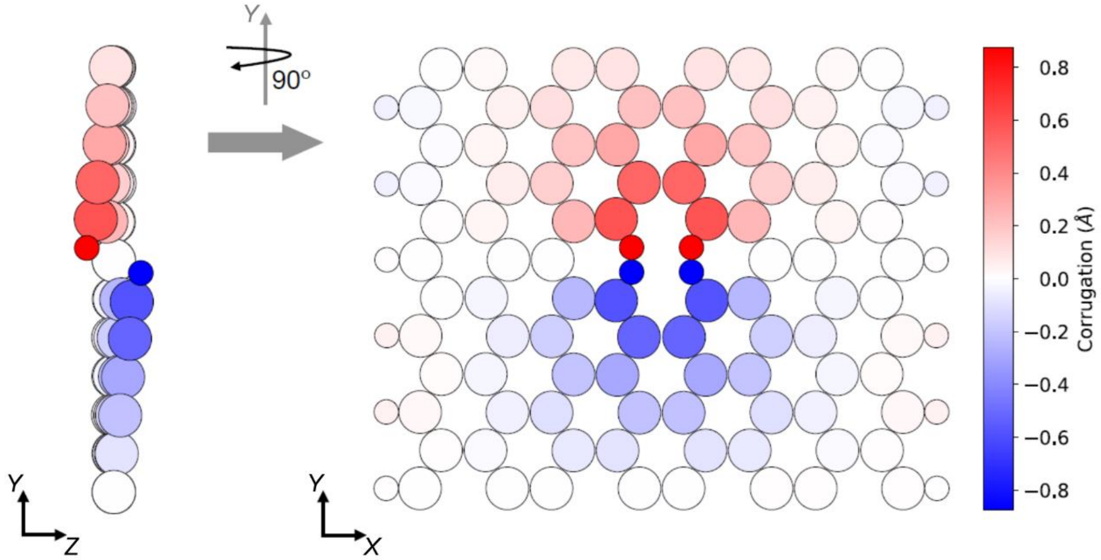

Supplementary Figure 27: **Corrugated divacancy type GNRs**. Colormap highlighting the corrugation of the GNRs with periodically spaced divacancies. Divacancies are non planar pores[2].

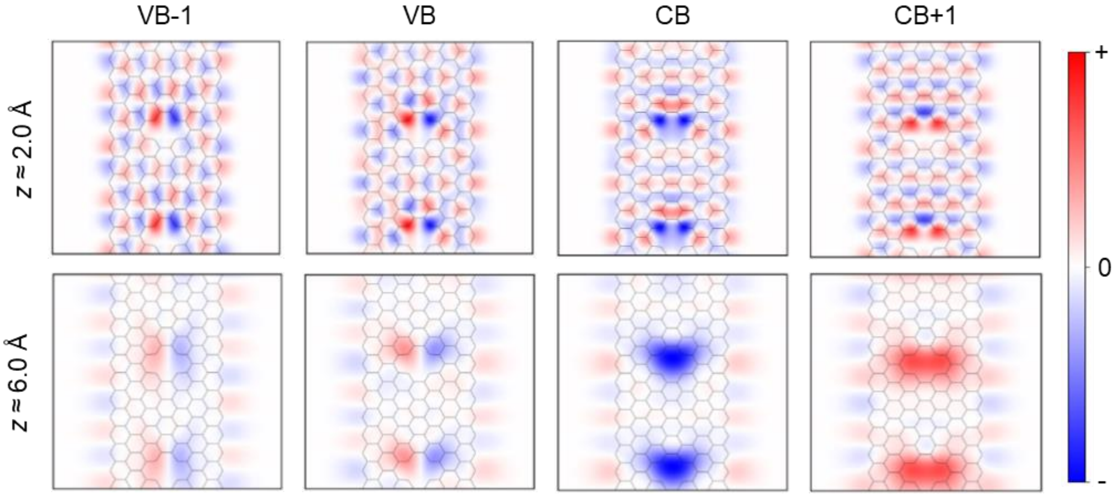

Supplementary Figure 28: **Effect of tip-sample distance on the electronic orbital decay of GNRs with periodically spaced divacancies**. Kohn-Sham orbitals of VB-1, VB, CB, CB+1 at the band onsets (Γ-point) of a GNR evaluated from 2 Å to 6 Å.

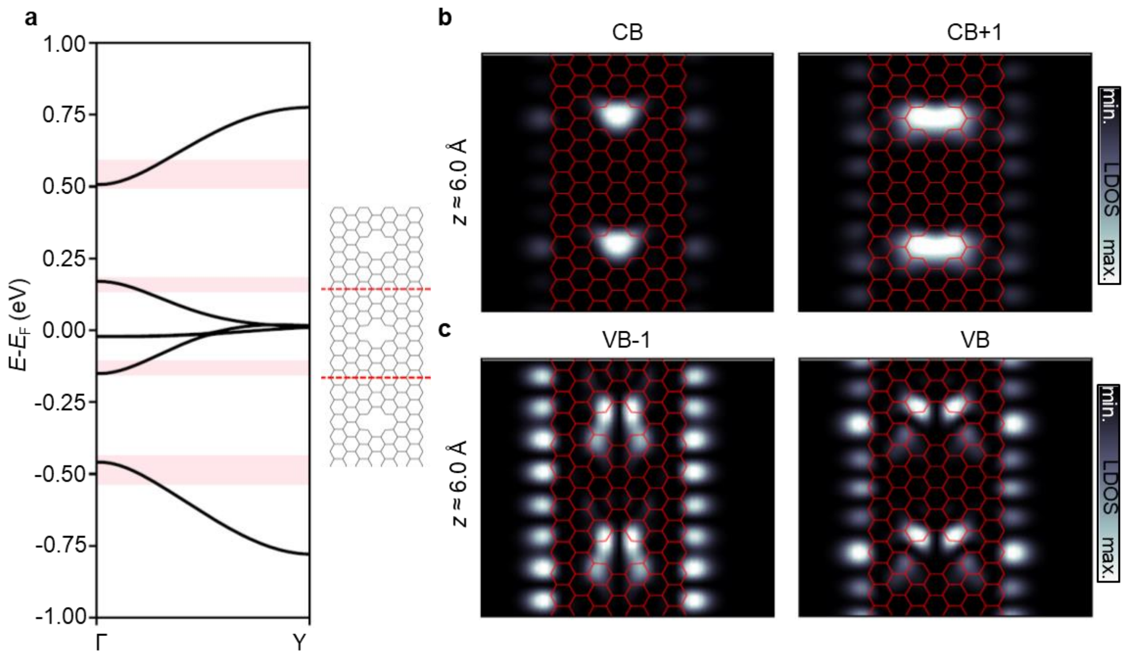

Supplementary Figure 29: **LDOS simulations of divacancy type GNRs**. Band structure (a) and LDOS map simulations (b,c) of GNRs with periodically spaced divacancies (evaluated at 6 Å away from the GNR). The electron density decays and is confined into the GNR edges and divacancies.

Supplementary References

1. Söde, H. *et al.* Electronic band dispersion of graphene nanoribbons via Fourier-transformed scanning tunneling spectroscopy. *Phys. Rev. B* **91**, 045429 (2015).
2. Yin, R. *et al.* Step-Assisted On-Surface Synthesis of Graphene Nanoribbons Embedded with Periodic Divacancies. *J. Am. Chem. Soc.* **144**, 14798–14808 (2022).
